# Catharsis in Gynecology

**Published:** 1899-03

**Authors:** 


### CATHARSIS IN GYNECOLOGY.

In the January issue of the *New England Medical Monthly* Dr. Denslow Lewis, Professor of Gynecology in the Chicago Polyclinic and otherwise distinguished as an obstetrician, has touched upon a theme of great interest not only to the obstetrical specialist but even of greater advantage to the general practitioner. His scholarly paper is entitled "The Treatment of Ambulatory Gynecologic Cases," and is the record of an extensive practice which Dr. Lewis has honored for twenty years. As noted it will appeal strongly to the specialist, and to them there is no need to call their attention now to the paper.

But the general practitioners may have overlooked the article through the mistaken assumption that it concerned only the specialist. To convince them of their error will be easy. All that is needed is to cite one of the topics on which the brilliant author gives the results of his long and complicated experience. Every medical man has learned to dread in his treatment of confinement cases the symptomatic obstruction of regular and thorough peristalsis. At all times it is safe to groan, "Constipation, thy name is woman." But parturition presents this physical predisposition of woman in not only its annoying but in its most grave and threatening aspects. Always a menace, it then becomes a source of great danger; in the most satisfactory cases on other accounts it stands ready to produce dangerous complications, and in every case which presents other difficulties this element is at hand to introduce a new set of complications. The paper proceeds to recite the great remedial agent which Dr. Lewis has found most successful in securing the greatly desired freedom of catharsis and systematic regularity of intestinal peristalsia. His reliance is based on a regular administration of a cathartic which avoids the element of drastic action. For all of twenty years he has exhibited cascara sagrada and he has found in that practice the reliable quality of Park, Davis and Co.'s preparation. It often happens that special needs of individual cases have made it proper or obligatory to unite with the cascara sagrada other cathartic principles, but this has always been the element on which he has relied to form the basis of a most successful treatment. Where an effect is reached by means so simple and easily attainable, the general practitioner will find himself just as well equipped as the specialist to deal with this ever-present complication of childbirth.

